# Longitudinal Changes in Component Processes of Working Memory

**DOI:** 10.1523/ENEURO.0052-17.2017

**Published:** 2017-03-20

**Authors:** Anna Rieckmann, Sara Pudas, Lars Nyberg

**Affiliations:** 1 Department of Radiation Sciences, Umeå University, 901 87 Umeå, Sweden; 2 Center for Functional Brain Imaging, Umeå University, 901 87 Umeå, Sweden; 3 Department of Integrative Medical Biology, Umeå University, 901 87 Umeå, Sweden

**Keywords:** Working memory, fMRI, aging, longitudinal, fronto-parietal

## Abstract

Working memory (WM) entails maintenance and manipulation of information in the absence of sensory input. This study investigated the trajectories and neural basis of these component processes of WM functions in aging. Longitudinal human functional magnetic resonance imaging (fMRI) data are presented from 136 older individuals (55–80 years) who were scanned at baseline and again 4 years later. We obtained evidence that age-related changes in parietal and frontal components of the WM core network are dissociable in terms of their role in maintenance of perceptual representations and further manipulation of this information, respectively. Individual difference analyses in performance subgroups showed that only prefrontal changes in fMRI activation were accompanied by changes in performance, but parietal brain activity was related to study dropout. We discuss the results in terms of possible neurobiological causes underlying separable aging-related declines in inferior parietal cortex and lateral prefrontal cortex that differentially affect WM functions.

## Significance Statement

Working memory (WM) describes the ability to maintain and manipulate information over brief periods of time after the information is no longer present in the environment, which is important for human goal-oriented behavior, reasoning, and decision-making. Age-related changes in WM functions and their neural basis are not fully understood, largely because of a scarcity of longitudinal data. Using functional MRI, this study of 136 older adults provides novel evidence for a decline of WM functions and underlying brain activity over a 4-year interval. We suggest the existence of two separable, age-related changes in brain function that differentially affect WM functions.

## Introduction

Working memory (WM) is an emergent property of interactions among core cognitive processes that serves short-term maintenance and manipulation of information in the absence of sensory input (Baddeley, 2003; Eriksson et al., 2015). Sustained attention to internal memory representations is a key component process of maintenance and has been linked to a fronto-parietal network including medial prefrontal, dorsolateral prefrontal cortex (DLPFC), and lateral parietal and temporal areas (Eriksson et al., 2015; Nyberg and Eriksson, 2015). Operations that support manipulation of the content in WM include mental arithmetic, alphabetical transformations, and chunking. Different brain networks are engaged for specific manipulation operations, but a general role for mid-DLPFC has been recognized (Eriksson et al., 2015; Nyberg and Eriksson, 2015).

Age-related changes in component processes of WM and their neural basis are not fully understood, largely because of a scarcity of longitudinal data. There is suggestive evidence from cross-sectional brain imaging studies that older adults need to recruit the fronto-parietal network to a higher degree than younger adults to maintain information in WM at the same task load (Mattay et al., 2006; Nyberg et al., 2009, 2014; Cappell et al., 2010), possibly reflecting greater attentional demands for older adults (Gazzaley et al., 2005; McNab et al., 2015). During cognitively demanding tasks that include manipulation of information, older adults recruit the DLPFC to a lower degree, which could reflect aging-related neurobiological changes in this region (Mattay et al., 2006; Nagel et al., 2009; Nyberg et al., 2009, 2014; Cappell et al., 2010; Reuter-Lorenz et al., 2010).

The present study, for the first time, examined longitudinal changes in WM and corresponding brain activity during a task in which one condition taxed WM maintenance only and another required both maintenance and manipulation of the stimuli (Chee and Choo, 2004; Pudas et al., 2009; Nyberg et al., 2014). Based on the available cross-sectional evidence, we predicted opposing age changes for maintenance and manipulation: aging-related increases were expected for fronto-parietal regions involved in maintenance, and thus in both WM conditions, and aging-related decreases were expected for frontal regions involved in manipulation, and thus only in the WM manipulation condition.

Because previous studies indicate marked heterogeneity in how aging influences cognition (Christensen et al., 1999; Habib et al., 2007; Josefsson et al., 2012) and brain activity (Nagel et al., 2009; Nyberg et al., 2009), we further extend our analyses in two important ways. (1) Subgroups of participants that differed in longitudinal WM performance change (decliners vs. stable) were analyzed separately. In keeping with our predictions that increasing activity during maintenance and decreasing activity during manipulation are reflective of a failing WM system in aging, we expected this pattern to be pronounced for decliners compared with those individuals that remain stable. (2) Brain activity at the first session was analyzed separately for participants who returned versus dropped out at the second scanning session. Behavioral studies have shown that dropout causes a positive bias because those remaining in the study tend to be performing better than those not returning (e.g., Cooney et al., 1988; Dufouil et al., 2004; Josefsson et al., 2012). Under the assumption that individuals do not drop out at random but often due to impending health and cognitive problems, we predict dropouts to show increased activity during maintenance and decreased activity during manipulation already at baseline, compared with individuals that remained in the study for the next 4 years.

## Materials and Methods

### Participants

Data in the present study come from the longitudinal, population-based study BETULA (Nilsson et al., 1997) and included 217 adults between ages 55 and 80 who fulfilled inclusion criteria (i.e., no contraindications to MRI or notable artifacts in the MRI acquisition, no history of neurologic or psychiatric disease, no dementia) and who completed a functional MRI (fMRI) scan at one measurement point. 136 of these individuals returned for a second fMRI scan 4 years later, from here on referred to as “returners” (mean age 64.98 years, SD 6.81; mean education 13.47 years, SD 4.00; 62 females), compared with the “dropouts” (*n* = 81; mean age 67.45 years, SD 8.12; mean education 13.11 years, SD 4.19; 19 females). When contacted for the follow-up scan, the majority of the dropouts (*n* = 40) reported health-related reasons for their refusal to participate in the second scan, including lack of energy, discomfort, MRI contraindications, and death. Only nine participants were not able to participate because of lack of time or relocation. The remaining 32 dropouts did not give a reason for ending their study participation.

The main focus of this article is on the returners and longitudinal changes in fMRI activation and performance. Data from the dropouts is presented where we consider them important for the interpretation of results in the returners. The participants in this study are a subsample of individuals previously analyzed for cross-sectional effects (Nyberg et al., 2014).

### WM task

During fMRI acquisition, participants performed a working memory task that included maintenance, manipulation, and control conditions. In the maintenance condition, participants were shown four target letters at a time for 2 s, followed by a fixation star for 3.5 s. A probe letter was then shown for 2.5 s, and participants were asked to indicate whether the probe letter was one of the four target letters. The manipulation condition had the same timing and design, but only two target letters were shown to the participants. Their task was to indicate whether the probe letter was the subsequent letter in the alphabet to any of the two target letters, thus requiring maintenance and manipulation of the to-be-remembered information. The control condition was comparable to the maintenance condition but included four identical target letters so that participants needed to maintain only one letter in WM. All target letters were presented in lowercase and all probe letters in capitals to decrease memorization purely based on visual representation. The task was divided into six blocks of each condition, and each block included three trials and lasted 27 s. To evaluate an individual’s performance on the in-scanner task, the number of hits minus false alarms was computed for each condition at each measurement point.

### Performance subgroups

To test whether changes in brain activity were related to task performance, the difference score between manipulation and maintenance performance on the in-scanner task was used to generate two performance subgroups. Because both of the key conditions of the task demand maintenance of information, a difference score was used to capture the processes specific to the WM manipulation operation. (Superscript letters listed with *p*-values correspond to the statistical tests shown in Table 4.) Two groups of 50 participants were selected to match in terms of baseline performance (group 1, mean –0.80, SD 0.95; group 2, mean –0.90, SD 1.06; *t*_(98)_ = 0.50, *p* = 0.62^a^), baseline age (group 1, mean 64.16, SD 0.62; group 2, mean 63.81, SD = 6.29; *t*_(98)_ = 0.28, *p* = 0.78^c^), and education (group 1, mean 13.40, SD 4.49; group 2, mean 14.04, SD 3.36; *t*_(98)_ = –0.81, *p* = 0.42^d^) but to show different trajectories of performance over time. At follow-up, group 1 showed significantly lower performance than group 2 (group 1, mean –2.28, SD 1.11; group 2, mean –0.12, SD 0.96; *t*_(98)_ = –10.42, *p* < 0.01^b^; Fig. 3*C*).

To further validate that the subgrouping captured differences in decline of WM functions, an offline *n*-back task was used to assess updating of information outside the scanner. A list of 40 words was presented visually one at a time, at a rate of one word per 3 s. Participants were instructed to say “yes” if the current word also occurred two words back in the list and “no” if the current word was not the same as the word presented two words back. The sum of correct responses was recorded as the behavioral measure of interest.

The *n*-back task confirmed that groups 1 and 2 did not differ in WM performance at baseline (group 1, mean 31.28, SD 2.98; group 2, mean 31.94, SD 5.03; *t*_(98)_ = –0.79, *p* = 0.43^e^) but that differences emerged at follow-up (group 1, mean 32.81, SD 4.16; group 2, mean 34.29, SD 2.96; *t*_(98)_ = –2.01, *p* = 0.05^f^). Groups 1 and 2 will be referred to as “decliners” and “stable” subgroups, respectively, but it should be noted that the stable subgroup actually showed an increase in performance, likely because of practice effects.

### MRI acquisition

MRI data were acquired on a 3T-GE MRI scanner and included a structural T1-weighted MRI scan and the fMRI run. The fMRI gradient-echo-planar imaging sequence lasted ∼10 min and collected a total of 290 volumes with the following parameters: TR = 2000 ms, TE = 30 ms, flip angle = 80°, field of view = 25 cm, and 37 transaxial slices of 3.4 mm (0.5 mm gap). Ten dummy scans were collected to allow for the fMRI signal to reach equilibration. The stimuli were presented on a computer screen seen through a tilted mirror. E-Prime (Psychology Software Tools) was used for stimulus presentation and recording of responses from the response pad.

### fMRI analyses

Preprocessing of the fMRI data included slice-timing correction, movement correction by unwarping and realignment to the first image of each volume, and normalization of each scan from each time point to a sample-specific template that included information from the returners, from both measurement points (DARTEL; Ashburner, 2007). Data were resliced and aligned to 2 × 2 × 2 Montreal Neurologic Institute standard space and smoothed with an 8-mm full width at half maximum Gaussian kernel.

The first-level analysis was performed separately for each scan at each time point. The data were high-pass filtered (128 s), and a general linear model was set up to include regressors for each condition, convolved with a canonical hemodynamic response function. Contrasts of interest were then set up for (1) manipulation–maintenance, (2) manipulation–control, and (3) maintenance–control. Six realignment parameters were included as covariates of no interest to remove movement-related artifacts.

Statistical tests are summarized in Table 4. Time-dependent changes in fMRI activation were evaluated with a whole-brain voxelwise paired *t*-test (baseline vs. follow-up) for the two contrast images from the first level. A second-level conjunction analysis was used to determine if there existed “process-general” changes across time (follow-up – baseline). In addition, for all analyses, difference images of the two time points were entered into a multiple regression analysis with covariates of interest for age and (age)^2^, to address whether a change in activation over time differed depending on the person’s baseline age. For illustration only, group mean activation maps were generated at baseline.

For comparison to the longitudinal effect, a cross-sectional multiple regression analysis with age as the covariate of interest was also computed for the contrasts of interest at baseline. All voxelwise analyses were performed in SPM12 and evaluated at *p* < 0.05, with a family-wise error correction. Where we considered it meaningful, results are also reported at a liberal threshold of *p* < 0.0001.

To generate bar graphs for illustration and perform post hoc *t*-tests and regression analyses to explore time, condition, and group comparisons, a 5-mm spheric mask was centered on peak activations, and the average signal within the mask was extracted for each participant from the respective first-level parameter estimates (β) for a particular contrast. In a post hoc comparison of dropouts versus returners, the predictive power of contrast values in regions of interest at baseline were evaluated against a measure of global (whole brain) atrophy. Whole-brain volume (gray and white matter) was computed after an automated cortical reconstruction and volumetric segmentation of a T1-weighted MRI image with the Freesurfer image analysis suite (Fischl et al., 2002). Whole-brain volume was adjusted for estimated total intracranial volume, following the methods described in Buckner et al., 2004, to derive a measure of global atrophy.

Behavioral analyses and post hoc tests on first-level contrast values were conducted using SPSS (v 21) and R (v 3.1.3).

## Results

The longitudinal analysis of brain activation during WM revealed both condition-general increases in posterior parts of the maintenance network and decreases specific to the manipulation condition in anterior parts over a period of 4 years. Cross-sectional multiple regression analyses did not reveal these age-related patterns (*p* > 0.0001^g1,h1,i1^, uncorrected), suggesting that longitudinal analyses of component processes of WM are sensitive to subtle age-related changes that are not revealed in cross-sectional comparisons of the same individuals.

### Age-related increases in posterior parts of the maintenance network

**Figure 1.**
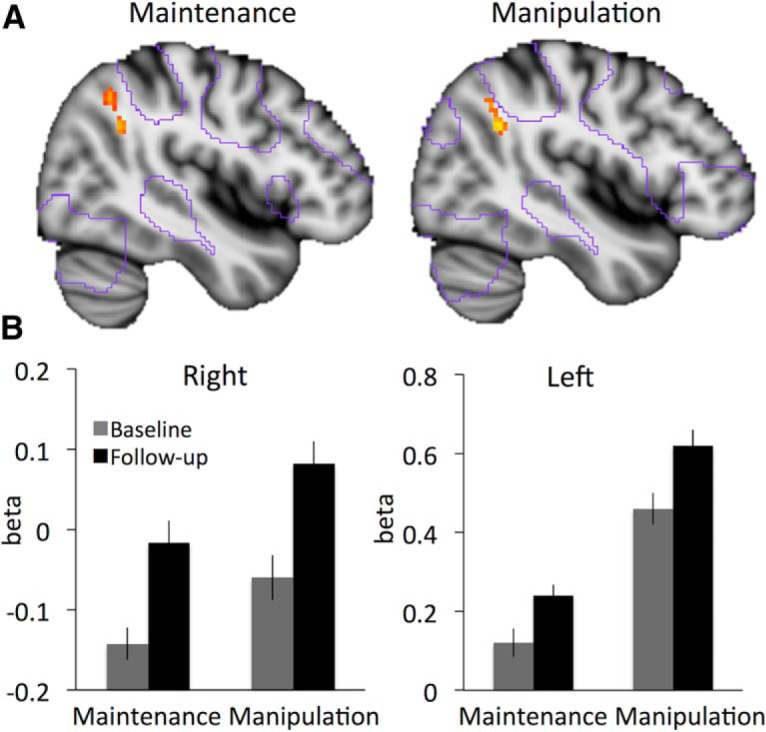
Condition-general longitudinal increase in parietal activity over 4 years. ***A***, Changes in brain activity over 4 years are observed in right inferior parietal cortex for maintenance and manipulation. Purple outlines, areas that were activate at baseline; red-yellow colors, significant increases (*p* < 0.05, FWE corrected). ***B***, Corresponding bar graph for right and left inferior parietal cortex are based on peak activity in this region identified in a conjunction analysis of both conditions; cf. Table 1 for cluster statistics.

In both critical contrasts (manipulation–control; maintenance–control), time-dependent increases were observed in right inferior parietal cortex (angular gyrus, BA 39; Fig. 1*A*; *p* < 0.05^g2,h2^, corrected). A conjunction analysis confirmed significant^j^ common activations in this area (Table 1, Fig. 1*B*). A second significant cluster of overlapping activation was found in left temporal cortex but was not inspected further because of its small spatial extent (three voxels). There were no significant^i2^ increases specific to one condition (i.e., manipulation vs. maintenance), which supported our expectation that aging is associated with increasing levels of brain activity during both conditions, as both conditions tax WM maintenance.

**Table 1.** Peak loci of activation changes (increases and decreases)

Contrast	Location	*T*	*k*	Threshold
Condition-general (manipulation and maintenance)				
Increases	44 –52 30 (right parietal cortex)*	4.91	38	<0.05†
	–54 –52 –18 (left temporal cortex)	4.56	3	<0.05†
	–32 –62 34 (left parietal cortex)*	4.38	59	<0.0001
Decreases	—			Not significant
Condition-specific (manipulation–maintenance)				
Increases	—			Not significant
Decreases	–46 28 22 (lateral PFC) *	4.20	440	<0.0001‡

* Activation from these clusters are used in post hoc analyses (main text).

† Corrected family-wise error rate at voxel level.

‡ Corrected family-wise error rate at cluster level .

As indicated by contours of the activation from baseline Fig. 1*A*, the increases were observed at the borders of the core working memory network, which includes anterior, dorsolateral, and dorsomedial prefrontal cortex, as well as inferior lateral parietal areas, suggesting that with aging a larger parietal area was engaged during maintenance. It should be noted that at a less conservative threshold of *p* < 0.0001^j2^ (uncorrected, Table 1), increases in inferior parietal activation over time were observed also in left parietal cortex. Extracted beta values from right and left parietal cortex (follow-up – baseline) correlated highly for both maintenance (*r* = .83, *p* < 0.01^k^) and manipulation (*r* = .68, *p* < 0.01^l^), suggesting that the effect was pronounced in the right hemisphere but not strictly lateralized. For further analyses presented below, the β estimates from right and left parietal increases were therefore averaged across hemispheres.

Increases in activity did not differ significantly^g3,h3,i3^, depending on an individual’s baseline age, suggesting linear changes in activation across the age range of the sample.

### Age-related decreases in the DLPFC during WM manipulation

**Figure 2.**
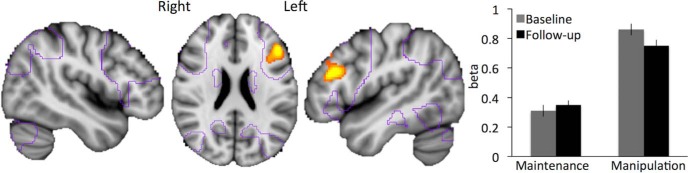
Condition-specific (manipulation–maintenance) decrease in brain activity over 4 years. Purple outlines, areas that were active at baseline; red-yellow color, significant decreases in left prefrontal cortex (*p* < 0.0001, uncorrected). Corresponding bar graphs for left lateral prefrontal cortex are based on peak activity in this region; cf. Table 1.

A comparison of the contrast between conditions (manipulation–maintenance) over time showed activation decreases in a cluster spanning the left lateral prefrontal cortex (including DLPFC/middle frontal gyrus/BA 46 and parts of the inferior frontal gyrus/BA 45; Table 1, Fig. 2). It should be noted that peak voxel activity did not survive correction for multiple comparisons (it was significant at *p* < 0.0001^i2^, uncorrected; Table 1) but the probability that a cluster of that size would occur by chance in these data were *p* < 0.05, increasing our confidence in this result (i.e., corrected at cluster level).

In Fig. 2, contours of the activation from baseline illustrate that the cluster was in the lateral PFC area of the core WM network. The corresponding bar plot in Fig. 2 shows that activation decreases were driven by reduced recruitment of DLPFC during the manipulation condition (relative to the control condition, *t*_(135)_ = 2.50; *p* = 0.01^m^). An increase in activity during the maintenance condition (relative to the control condition) was observed over time, but in post hoc comparisons it was not significant (*t*_(135)_ = –0.97, *p* = 0.33^n^). This pattern provides longitudinal support for the hypothesis that aging is characterized by reduced DLPFC recruitment specifically during WM manipulation. There were no significant time-dependent increases in fMRI activation for this contrast (manipulation–maintenance) and no brain regions in which time-dependent changes in either direction differed depending on an individuals’ baseline age.

Across individuals, there was no significant association between the maintenance-related increase in bilateral parietal cortex recruitment and the decrease in left DLPFC recruitment for manipulation (*r* = 0.11, *p* = 0.20^°^). This indicates that the time-dependent changes in DLPFC and parietal cortex are separable events, and individual difference analysis further explored factors that correlate with these different changes in brain activity.

### Individual differences

We first examined whether variability in changes of brain activity related to changes in task performance. The behavioral analyses showed that participants increased their performance over time for the maintenance condition and decreased performance on the manipulation condition (ANOVA time × condition: *F*_(1135)_ = 4.82, *p* = 0.03^p^; Table 2). This prompted the hypothesis that the declining activation in left DLPFC specific for manipulation (– maintenance) would be related to the specific decrease in manipulation task performance. For this test, performance for the manipulation trials (– maintenance) was used to select two performance subgroups of 50 participants each who did not differ in terms of baseline performance (Fig. 4*A*; see Materials and Methods for details). Fig. 4*B* shows that only individuals with subsequent performance decline showed significantly decreased recruitment of the DLPFC at follow-up (*t*_(49)_ = 3.09, *p* < 0.01^r^; cf. stable group *t*_(49)_ = 0.54, *p* = 0.59^s^). This effect appeared specific to the changes in DLPFC, as the condition-general parietal increases did not distinguish the performance subgroups. (Significant time (1 | 2) × region (left PFC|bilateral parietal) × group (decliners|stable) interaction: *F*_(1,98)_ = 5.63, *p* = 0.02^q^).

**Table 2.** Task performance (hits – false alarms and SD) by condition

	Condition		
Task	Control	Maintenance	Manipulation
Baseline (*n* = 136)	8.63 (0.63)	8.37 (0.81)	7.35 (1.45)
Follow-up (*n* = 136)	8.70 (0.65)	8.41 (0.94)	7.08 (1.44)
Dropouts at baseline (*n* = 81)	8.57 (0.10)	8.12 (0.12)	6.77 (0.16)

**Figure 3.**
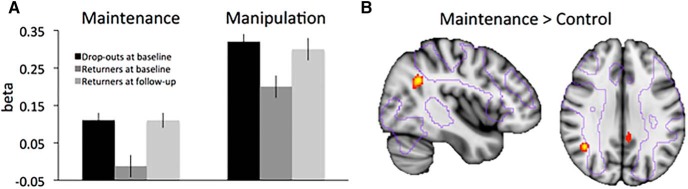
Dropout analysis. Increased levels of brain activity in right inferior parietal cortex (β values from the cluster identified in Table 1) for dropouts at baseline (***A***). Voxelwise analysis of higher brain activity during the maintenance condition in dropouts > returners shown in red-yellow (*p* < 0.0001, uncorrected for illustration), overlaid onto the purple outline of the brain areas implicated in maintenance in the dropouts.

Next, we examined levels of brain activity in the regions of interest derived from the longitudinal analysis for the dropouts at baseline (i.e., the 81 individuals who did not return for the follow-up session). Interestingly, dropouts showed significantly greater recruitment of bilateral inferior parietal cortex already at baseline, compared with the individuals who remained in the study (Fig. 3; maintenance: *t*_(215)_ = 3.59, *p* < 0.01^t^; manipulation: *t*_(215)_ = –2.70, *p* = 0.01^u^). Two further analyses were designed to test the specificity of this finding. A voxelwise whole-brain comparison between dropouts and returners at baseline showed that higher levels of brain activity in dropouts were indeed significant only in the inferior parietal lobe (*x* = 40, *y* = –54, *z* = 28, at *p* < 0.05^g4,h4^, FWE corrected for multiple comparisons for the contrast maintenance > baseline). At a more lenient threshold of *p* < 0.0001 (uncorrected), greater levels of inferior parietal activity in the dropouts were observed bilaterally and for both conditions. A logistic regression with dropout at follow-up (yes/no) as the dependent variable then further confirmed that greater levels of inferior parietal activation at baseline strongly predicted dropout, even when controlling for DLPFC activation, global brain atrophy, performance on the task, sex, and baseline age (Table 3). This means that for an individual with right parietal activation 1SD above the sample mean at baseline, the odds of not returning for the follow-up scan increased by 70%^v^. Of note, being a woman and performance in the manipulation task at baseline were also significant, independent, predictors of dropout in the model.

**Table 3.** Predictors of later dropout

Variable	OR	SE	*p*
Bilateral parietal activation	1.69	0.17	<0.01
DLPFC activation	0.96	0.16	0.79
Global brain atrophy	0.81	0.18	0.24
Sex (reference category male)	1.95	0.31	0.03
Manipulation performance (hits – false alarms)	0.79	0.02	0.03
Age (years)	1.02	0.02	0.37

Dependent variable, dropout (yes/no). Predictors: bilateral parietal activation, *z*-scored baseline activation, averaged across manipulation and maintenance; DLPFC activation, *z*-scored baseline activation; global brain atrophy, global brain volum, *z*-scored, corrected for intracranial volume.

**Figure 4.**
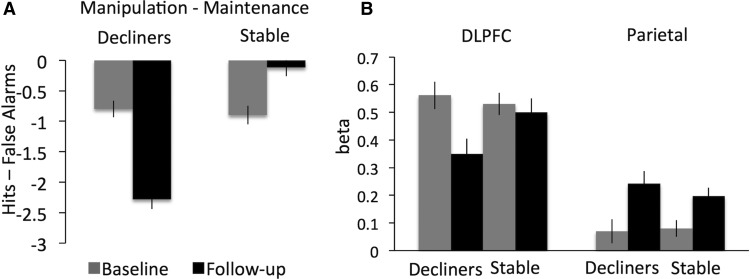
Subgroup analyses. ***A***, WM performance (hits – false alarms, manipulation–maintenance) by time point for subgroups decliners (*n* = 50) and stable (*n* = 50). ***B***, fMRI activation (β) by subgroup, brain region, and time.

**Table 4.** Statistical table

Line	Data/dependent variable	Type of test	Statistic	Confidence
	Methods: performance subgroups			
a	Hits – false alarms baseline (manipulation–maintenance)	*t*-test	*t* = 0.50; DoF = 98	*p* = 0.62; CI = –0.30/0.50
b	Hits – false alarms follow-up (manipulation–maintenance)	*t*-test	*t* = –10.42; DoF = 98	*p* < 0.001; CI = –2.57/–1.75
c	Baseline age (years)	*t*-test	*t* = 0.28; DoF = 98	*p* = 0.78; CI = –2.12/2.81
d	Education	*t*-test	*t* = –0.65; DoF = 98	*p* = 0.52; CI = –2.73/1.40
e	Sum of correct responses *n*-back, baseline	*t*-test	*t* = –0.79; DoF = 98	*p* = 0.43; CI = –2.31/0.99
f	Sum of correct responses *n*-back, follow-up	*t*-test	*t* = –2.01; DoF = 98	*p* = 0.05; CI = –2.94/–0.02
	Results: FMRI second level voxelwise analyses using SPM12			
g1	Contrast values, maintenance–control	Multiple regression at baseline (cross-sectional age effect)	*p* > 0.0001, no significant clusters	
g2		Paired *t*-test, baseline to follow-up (longitudinal effect)	*t* = 5.21; DoF = 135	*p* = 0.006^corrected^
g3		Multiple regression (time × age)	No significant clusters	
g4		*t*-test (dropout vs. returners)	*t* = 4.65	*p* < 0.03^corrected^
h1	Contrast values, manipulation–control	Multiple regression at baseline (cross-sectional age effect)	*p* > 0.0001, no significant clusters	
h2		Paired *t*-test, baseline to follow-up (longitudinal effect)	*t* = 5.11 DoF = 135	*p* = 0.009^corrected^
h3		Multiple regression (time × age)	No significant clusters	
h4		*t*-test (dropout vs. returners)	*t* > 3.13	P < 0.001^uncorrected^
i1	Contrast values, manipulation–maintenance	Multiple regression at baseline (cross-sectional age effect)	*p* > 0.0001, no significant clusters	
i2		Paired *t*-test, baseline to follow-up (longitudinal effect)	cf. Table 1	
i3		Multiple regression (time × age)	No significant clusters	
j	Contrast values, (1) maintenance–control; (2) manipulation–control	Conjunction	cf. Table 1	
	Results: post hoc/individual difference analyses			
k	Right and left parietal β (maintenance–control, follow-up – baseline)	Pearson’s correlation	*r* = 0.83; DoF = 136	*p* < 0.01; CI = 0.58/0.92
l	Right and left parietal β (manipulation–control, follow-up – baseline)	Pearson’s correlation	*r* = 0.68; DoF = 136	*p* < 0.01; CI = 0.35/0.85
m	DLPFC β (manipulation–control)	Paired *t*-test	*t* = 2.50; DoF = 135	*p* = 0.01; CI = 0.02/0.19
*n*	DLPFC β (maintenance–control)	Paired *t*-test	*t* = 0.97; DoF = 135	*p* = 0.33; CI = –0.11/0.04
o	DLPFC β (manipulation–maintenance, follow-up – baseline), bilateral parietal beta (average manipulation and maintenance, follow-up – baseline)	Pearson’s correlation	*r* = 0.11; DoF = 136	*p* = 0.20; CI = –0.06/0.29
*p*	Performance (hits – false alarms) by condition	Two-way ANOVA	*F* = 4.82; DoF = 135	*p* = 0.03; partial η^2^ = 0.03
q	DLPFC β (manipulation–maintenance)	Three-way ANOVA	*F* = 5.63; DoF = 98	*p* = 0.02; partial η^2^ = 0.05
r		Paired *t*-test	*t* = 3.09; DoF = 49	*p* < 0.01; CI = 0.08/0.36
s		Paired *t*-test	*t* = 0.54; DoF = 49	*p* = 0.59; CI = –0.07/0.13
t	Bilateral parietal β (maintenance–control, baseline)	*t*-test	*t* = –3.59; DoF = 215	*p* < 0.01; CI = –0.21/–0.03
u	Bilateral parietal β (manipulation–control, baseline)	*t*-test	*t* = –2.70; DoF = 215	*p* = 0.01; CI = –0.19/–0.03
v	Outcome: dropout (yes/no)	Logistic regression	cf. Table 3	

In summary, these results show that emerging performance differences in individuals who remained in the study were related to altered recruitment of the left DLPFC over time, whereas dropout was predicted by abnormally high parietal activation at baseline.

## Discussion

We provide novel evidence for age-related changes in brain activity during component processes of WM that are captured within individuals over a period of 4 years. Increases in bilateral parietal cortex activation over the 4-year period were observed in both WM conditions. Declining recruitment of the left lateral PFC, a core region of the WM network, was observed specifically in the condition taxing WM manipulation.

Prior research has suggested that activation of parietal WM network areas, along with visual cortex (Chen et al., 2012; Lee et al., 2013), is sufficient for tasks that require the maintenance and storage of perceptual information from the environment (Pochon et al., 2001). Via sustained attention, perceptual representations of the stimuli may be maintained in a state of activation until the information is no longer needed, irrespective of the prospective task at hand. A recent cross-sectional study in more than 29,000 individuals across the lifespan suggests that older age is associated with a greater reliance on focused attention during the encoding period of a WM task (McNab et al., 2015), which may reflect the increased parietal activation we observe. In contrast, the DLPFC is likely recruited when the maintained information is used prospectively and requires further manipulation of abstract representations of the material (Pochon et al., 2001; Chen et al., 2012; Lee et al., 2013). In line with a process-specific role of parietal and lateral prefrontal areas in WM, Badre and D'Esposito (2007) demonstrated increased prefrontal activity as representations required for task performance became more abstract along with general activation in inferior parietal lobe not specific to the representational level.

### Age-related decreases in lateral prefrontal activity during WM manipulation accompany changes in performance

The “resource capacity” hypothesis predicts a failure to up-regulate prefrontal activity when a capacity limit is reached, i.e., with increasing WM demands (Mattay et al., 2006; Reuter-Lorenz and Cappell, 2008). Aging lowers this capacity limit because more neural units are required to maintain task performance. Our results confirm this hypothesis, at least in parts, for the first time with longitudinal data. The left lateral PFC showed decreases in activity during manipulation, but not during maintenance, and was part of a network of fronto-parietal regions that responded strongly to increasing task demands (manipulation–maintenance) at baseline. The analysis in performance subgroups further suggested that aging-related changes in PFC activation are not uniform across individuals but pronounced in those who decline in performance.

However, in comparison to previous cross-sectional studies, here we find little evidence for prefrontal increases in individuals who successfully maintain task performance over time, and which may be interpreted to reflect “compensatory” activation during WM. Rather, individuals who showed stable performance over 4 years also showed stable lateral PFC activation over 4 years, consistent with the notion of brain maintenance as a determinant of successful aging (Nyberg et al., 2012).

One potential factor that might determine individual differences in prefrontal up-regulation during aging is age-related decline in dopamine functions, in particular of the D1 receptor. Primate studies (Brozoski et al., 1979; Sawaguchi and Goldman-Rakic, 1991) have demonstrated that dopamine depletion in PFC selectively impaired WM in monkeys, and dopamine signaling in PFC is thought to stabilize neural representations in WM (Servan-Schreiber, 1990; Durstewitz et al., 2000). Human multimodal imaging has supported this hypothesis and demonstrated that lower dopamine D1 receptor densities as measured with positron emission tomography are associated with lower prefrontal up-regulation (Bäckman et al., 2011) and lower coupling between lateral prefrontal and parietal WM areas (Rieckmann et al., 2011). In addition, human genetic studies of a functional polymorphism in the gene for COMT, which regulates prefrontal dopamine reuptake, have found associations between genotype and prefrontal up-regulation during WM (Mier et al., 2009; Nyberg et al., 2014).

### Dropout is predicted by parietal brain activity

Although an age-related decline in PFC up-regulation during WM is a common finding also in cross-sectional comparisons (Mattay et al., 2006; Nagel et al., 2009; Nyberg et al., 2009, 2014; Cappell et al., 2010; Reuter-Lorenz et al., 2010), findings of task-general increases in parietal activation in older adults during WM are not a common observation. Notably, whereas previously reported cross-sectional analyses of the current task and sample had revealed age-related reductions in frontal up-regulation during manipulation, parietal increases for either maintenance or manipulation had not been observed (Nyberg et al., 2014). This is important because prior research in other cognitive domains has also suggested that intra- and interindividual estimates of aging-related changes do not always align (Nyberg et al., 2010). In particular, associations between preclinical markers of impending disease, such as amyloid burden or hypometabolism for Alzheimer’s disease, and cognition or other brain markers are often not significant or very small in cross-sectional associations (Hedden et al., 2013), but longitudinal studies prove sensitive to reveal these associations (Storandt et al., 2009; Rieckmann et al., 2016). Based on comparisons across studies, we entertain the hypothesis that diverging cross-sectional and longitudinal effects are more likely to be observed in tasks (and brain areas) that are sensitive to age-related pathology, because sampling bias in cross-sectional studies is more likely for these outcomes. Following this line of reasoning, our current results would suggest that parietal increases are indicative of impending disease, whereas prefrontal declines are reflective of a normal aging process (e.g., a reduction in dopamine receptors). Indeed, early stages of Alzheimer’s disease are associated with pronounced brain structural deficits that primarily target posterior regions, including the parietal lobes (e.g., Thompson et al., 2003; Head, 2004).

### Study limitations

One limitation of the current study is the inability to track aging-related changes over more than two time points. We draw conclusions about independent aging-related cascades based on an absence of change–change correlations (i.e., between parietal and prefrontal activation and between parietal activation and performance). However, it is possible that change–change correlations are not occurring in parallel, but rather that one change affects another change some years later. We hope that future studies with three or more time points will reveal these time-lagged associations that we are unable to show in the current study design.

Another design limitation of the current study concerns the use of a blocked design, which did not allow us to further separate each trial into the stimuli encoding delay and response phase. For future investigations, the use of an event-related design may be desirable, because modeling different cognitive operations with greater specifically may further aid our interpretation of process-specific roles of parietal and lateral prefrontal areas in WM.

Finally, we acknowledge that we were not able to track the reasons for dropout in greater detail. We interpret our data based on the information that the majority of dropouts did not return for the follow-up scan because their health or well-being had declined, but it is important to acknowledge that we are missing the reason for dropout for a large portion of the sample, that this is likely a heterogeneous group, and that we did not have sufficient power to analyze further subgroups of dropouts.

## Conclusions

Our study shows that aging is accompanied by changes in WM functions and their neural correlates. In both critical contrasts (manipulation–control; maintenance–control), time-dependent increases were observed in right inferior parietal cortex. A comparison of the contrast between conditions (manipulation–maintenance) over time showed activation decreases in the left prefrontal cortex. The results suggest that the parietal and frontal components of the frontal-parietal WM core network may be dissociable in terms of their role in maintenance of perceptual representations (parietal) and further manipulation of this information (prefrontal). Future longitudinal studies are required to disentangle the possible neurobiological causes underlying separable aging-related declines in inferior parietal cortex and lateral prefrontal cortex.

## Synthesis

Reviewing Editor: Lila Davachi, New York University Decisions are customarily a result of the Reviewing Editor and the peer reviewers coming together and discussing their recommendations until a consensus is reached. When revisions are invited, a fact-based synthesis statement explaining their decision and outlining what is needed to prepare a revision will be listed below. Note: If this manuscript was transferred from JNeurosci and a decision was made to accept the manuscript without peer review, a brief statement to this effect will instead be what is listed below.

This manuscript was transferred from JNeurosci and a decision was made to accept the manuscript without additional peer review.
